# A cross-sectional analysis of light at night, neighborhood sociodemographics and urinary 6-sulfatoxymelatonin concentrations: implications for the conduct of health studies

**DOI:** 10.1186/1476-072X-12-39

**Published:** 2013-08-30

**Authors:** Susan Hurley, David O Nelson, Erika Garcia, Robert Gunier, Andrew Hertz, Peggy Reynolds

**Affiliations:** 1 Cancer Prevention Institute of California, 2001 Center Street, Suite 700, Berkeley, CA 94704, USA; 2 Department of Health Research and Policy, Stanford University School of Medicine, Stanford, CA 94305, USA

**Keywords:** Circadian disruption, Light at night, Melatonin, aMT6s, Socioeconomic status, Women

## Abstract

**Background:**

There is accumulating evidence that circadian disruption, mediated by alterations in melatonin levels, may play an etiologic role in a wide variety of diseases. The degree to which light-at-night (LAN) and other factors can alter melatonin levels is not well-documented. Our primary objective was to evaluate the degree to which estimates of outdoor environmental LAN predict 6-sulftoxymelatonin (aMT6s), the primary urinary metabolite of melatonin. We also evaluated other potential behavioral, sociodemographic, and anthropomorphic predictors of aMT6s.

**Methods:**

Study participants consisted of 303 members of the California Teachers Study who provided a 24-hour urine specimen and completed a self-administered questionnaire in 2000. Urinary aMT6s was measured using the Bühlmann ELISA. Outdoor LAN levels were estimated from satellite imagery data obtained from the U.S. Defense Meteorological Satellite Program’s (DMSP) Operational Linescan System and assigned to study participants’ geocoded residential address. Information on other potential predictors of aMT6s was derived from self-administered surveys. Neighborhood socioeconomic status (SES) was based on U.S. Census block group data.

**Results:**

Lower aMT6s levels were significantly associated with older age, shorter nights, and residential locations in lower SES neighborhoods. Outdoor sources of LAN estimated using low-dynamic range DMSP data had insufficient variability across urban neighborhoods to evaluate. While high-dynamic range DMSP offered much better variability, it was not significantly associated with urinary aMT6s.

**Conclusions:**

Future health studies should utilize the high-dynamic range DMSP data and should consider other potential sources of circadian disruption associated with living in lower SES neighborhoods.

## Background

There is growing evidence that environmental light pollution may play an etiologic role in a variety of diseases, including depression, cardiovascular disease, and cancer [1-10]. Such health effects are largely thought to be mediated via circadian disruption driven by alterations in melatonin production and secretion [11,12]. Melatonin, an endogenous hormone produced by the pineal gland, is the primary hormonal modulator of circadian regulation in mammals and is strongly influenced by exposure to visible light, with levels peaking during the darkness of night [13]. Predictors of melatonin levels in humans generally have not yet been well-characterized [11,14-18]. While there is fairly strong evidence that indoor artificial light-at-night (LAN) is sufficient to suppress melatonin levels in humans, it is not known whether outdoor environmental light pollution is sufficient to exert a similar effect [1,3,19]. Fully understanding such predictors is a necessary prerequisite for implementing epidemiologic studies on this topic.

The primary objective of the current analyses was to evaluate the degree to which estimates of outdoor environmental light-at-night (LAN) predict levels of 6-sulfatoxymelatonin (aMT6s), the primary urinary metabolite of melatonin, among 303 California women. Secondarily, we evaluated whether selected anthropomorphic characteristics and behavioral and sociodemographic factors predict urinary concentrations of aMT6s.

## Results

Creatinine adjusted urinary aMT6s concentrations ranged from 0.58 ng/mg-creatinine to 102.06 ng/mg-creatinine with a mean of 20.66 ng/mg-creatinine, a standard deviation of 17.78 ng/mg-creatinine and an interquartile range of 17.74 ng/mg-creatinine.

The characteristics of the study population are summarized in Table 1. Similar to the entire CTS cohort, the study population is predominantly non-Hispanic white (85%) and middle-aged (mean age = 55 years). Despite arising from an occupational cohort that requires at least a 4-year college degree among its members, study participants lived in both high and low SES neighborhoods, although skewed towards higher SES neighborhoods. By design, study participants were oversampled from rural neighborhoods (60.4%).

**Table 1:** Characteristics of study population (n = 303)

**Characteristics**	**Distribution**
Age at urine collection, years (mean,standard deviation)	55.0 (11.9)
BMI at urine collection, kg/m^2^ (mean,standard deviation)	26.6 (5.8)
Height at CTS baseline survey, inches (mean,standard deviation)	65.0 (2.7)
Packyears of smoking (mean,standard deviation)	4.0 (10.0)
Pillyears of aspirin use (mean,standard deviation)	0.69 (2.0)
Coffee consumption, average grams/day (mean,standard deviation)	238.2 (184.7)
Coffee consumption, average times/day (mean,standard deviation)	0.97 (0.72)
Strenuous exercise, 3-years prior to baseline, hours/wk (mean,standard deviation)	1.6 (2.4)
Strenuous exercise, lifetime prior to baseline, hours/wk (mean,standard deviation)	2.2 (2.3)
Age at first full-term pregnancy, years (mean,standard deviation)	27.0 (4.9)
Parity, total number of live and still births (mean,standard deviation)	1.7 (1.4)
Race/ethnicity (n,%)	
Non-Hispanic white	258 (85.1)
Hispanic	18 (5.9)
Black	3 (1.0)
Asian & Pacific Islander	13 (4.3)
Other/unknown	11 (3.6)
Menopausal Status, at baseline(n,%)	
Postmenospausal	131 (43.2)
Premenopausal or Perimenopausal	147 (48.5)
Unknown	25 (8.3)
Alcohol Consumption, year prior to baseline (n, %)	
None	86 (28.4)
<20 g/day	176 (58.1)
≥20 g/day	27 (8.9)
Unknown	14 (4.6)
Tobacco Smoke Exposure, at baseline (n, %)	
None	61 (20.1)
Passive only	142 (46.9)
Former active smoker	86 (28.4)
Current active smoker	12 (4.0)
Unknown	2 (0.7)
Oral contraceptive use, at baseline (n, %)	
Never	81 (26.7)
Former	198 (65.4)
Current	16 (5.3)
Unknown	8 (2.6)
Antidepressant use, daily use for at least 2 months at baseline	
Yes	29 (9.6)
No	237 (78.2)
Unknown	37 (12.2)
Hormone therapy use, at baseline (n, %)	
Premenopausal	142 (46.9)
Peri/post-menopausal, never used	34 (11.22)
Peri/post-menopausal, prior use	16 (5.3)
Peri/post-menopasual, current use	71 (23.4)
Other/unknown	40 (13.2)
Regular aspirin use, years at baseline	
None	240 (79.2)
<1	11 (3.6)
1	2 (0.7)
2	4 (1.3)
3-4	7 (2.3)
5-9	6 (2.0)
10+	31 (10.2)
Unknown/missing	2 (0.7)
Regular aspirin use, days/week at baseline	
None	240 (79.2)
1-3	41 (13.5)
4-6	9 (3.0)
7	12 (4.0)
Unknown	1 (0.3)
Daily Use of Calcium Blockers, in 2000	
Yes	4 (1.3)
No	246 (81.2)
Unknown	53 (17.5)
Neighborhood Urbanization	
Urban	120 (39.6)
Rural	183 (60.4)
Hours of sleep/night	
<5 hours	3 (1.0)
5-6 hours	63 (20.8)
7-9 hours	226 (74.6)
10+ hours	4 (1.3)
Unknown	7 (2.3)
Neighborhood Socioeconomic Status (mean, range)	
Median Family Income ($)	76,782 (37,337-140,387)
Percent of population below poverty level	6.8 (0.0 – 37.6)
Percent of adults age 25+ with college degree	34.3 (1.5 – 79.0)
Percent of adults without a high school degree	14.6 (1.2 – 56.8)
Percent of adults employed in a professional occupation	44.3 (6.7-74.3)

**Figure 1:**
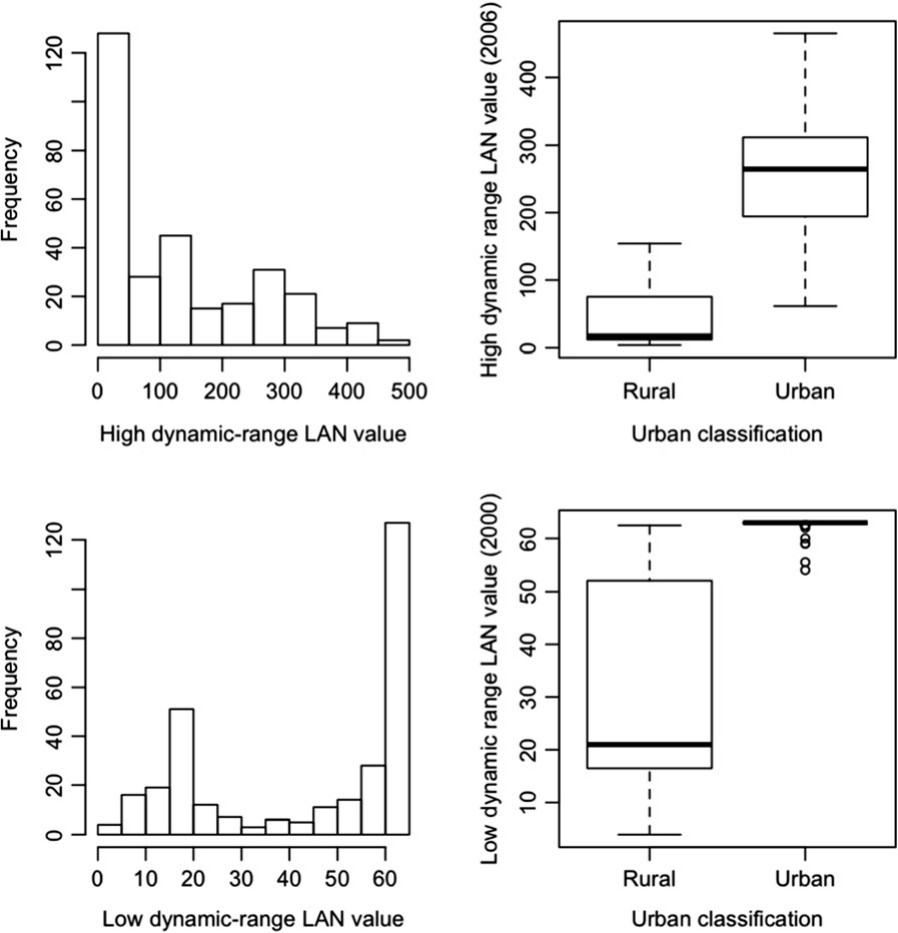
**Marginal distribution of Outdoor LAN estimates and distribution by urban classification for the low-dynamic range (year 2000) and the high-dynamic range (year 2006) data.** The units in the “low dynamic range” data are “DN” units, while the units in the “high dynamic range data” are scaled radiance units (see Methods).

Table 2 summarizes estimates of outdoor LAN exposures. The average length-of-night was 11 hours, reflecting the fact that most urine specimens were collected in the winter and early spring months. The radiance estimates of outdoor LAN ranged from 4 to 63, with a mean value of 43.4, for the low-dynamic range 2000 data, and 3.8 to 465.2, with a mean of 128.4, for the high-dynamic range 2006 data. Figure 1 illustrates the greater range and variability in radiance estimates offered by the high-dynamic range data. More importantly, Figure 1 illustrates that the low-dynamic range data lacks sufficient variability at the upper range to discriminate areas with higher LAN values within urban neighborhoods. In addition, LAN values were universally higher among women living in urban compared to rural neighborhoods, with no overlap in values across rural and urban neighborhoods. In contrast, the high-dynamic range data offers variability in values within both rural and urban areas as well as some overlap in values across urban and rural areas. Consequently, we chose to rely on the high-dynamic range data for the remainder of our analyses.

**Table 2:** Estimates of outdoor LAN among study participants (n = 303)

	**Mean (Range)**
Outdoor LAN satellite values (annual average radiance)	
Low-dynamic range, 2000 average (DN units)	43.4 (4 – 63)
High-dynamic range, 2006 average (scaled radiance units)	128.4 (3.8 – 465.2)
Length-of-night on day of urine collection (hours)	11 (9.2 – 14.4)

The results from our random forests analyses to identify the most important predictors of urinary aMT6s concentrations, based on the scaled variable importance values (mean divided by the standard deviation), identified the following 8 variables as potentially important predictors of aMT6s: hormone therapy use; oral contraceptive use; length-of-night; coffee consumption; strenuous exercise in the past 3 years; age; menopausal status; and neighborhood SES. Overall, however, none of these variables, individually or combined, explained a substantial proportion of the variability in measured aMT6s levels (*data not shown*).

**Figure 2:**
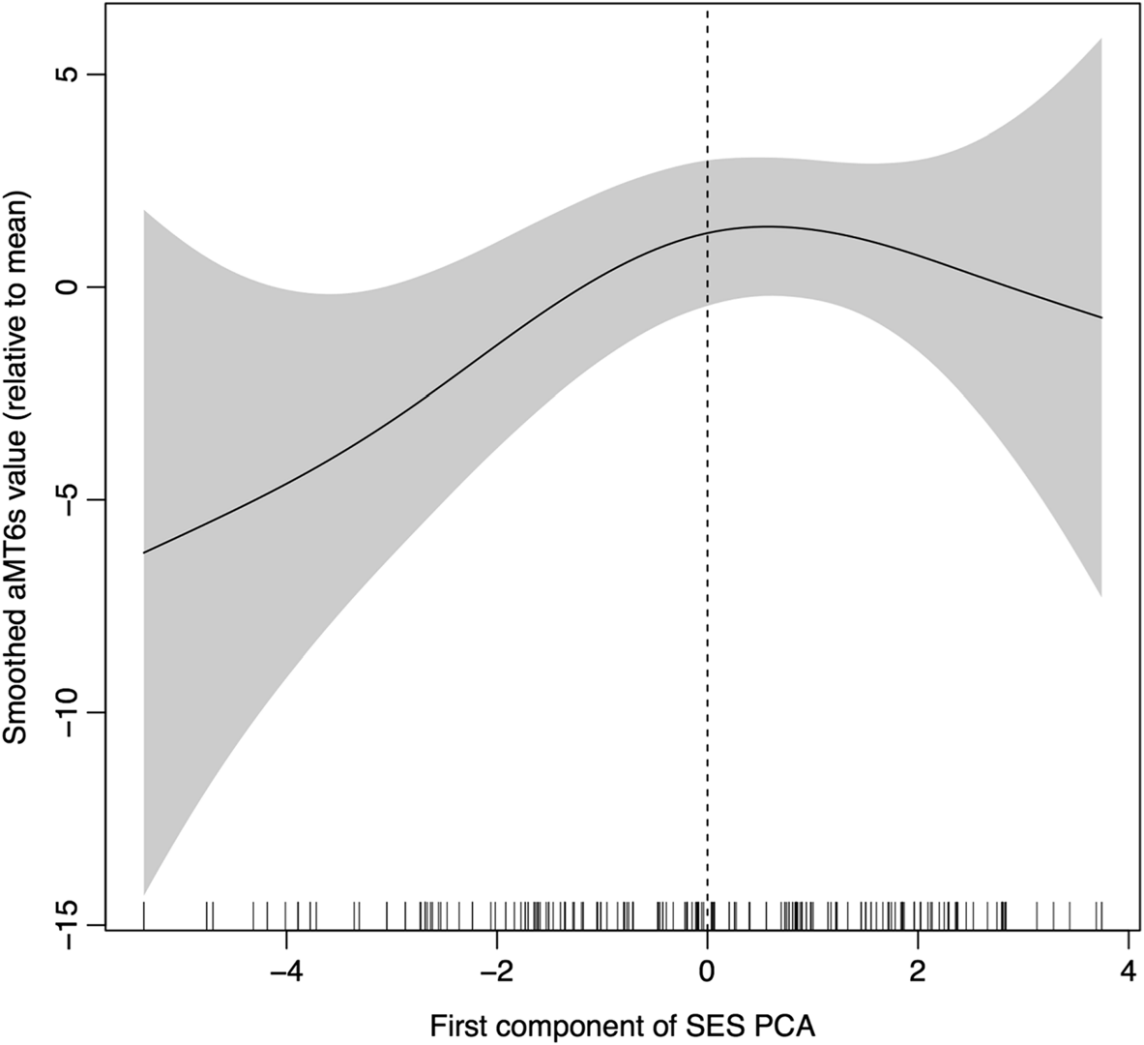
**Estimated relationship between neighborhood SES and aMT6s, along with the pointwise 95% confidence band (shaded in gray).** The relationship increases until SES equals zero, at which point it flattens out and no statistically significant slope remains (the dashed vertical line indicated where the SES PCA component is equal to zero). The lines across the bottom indicate the SES values for the sample. Note that the spline only shows deviation of aMT6s relative to the average aMT6s value across the sample.

Prior to conducting our regression analyses, we examined the shape of the relationship between SES and aMT6s. A plot of the smoothed aMT6s on the first component of our SES PCA (Figure 2) demonstrates that increases in melatonin are seen with increases in SES but only among those living in neighborhoods for which the PCA value for SES did not exceed zero. Once the summary neighborhood SES reaches the average value (represented by zero), the relationship flattens out. Consequently, in our regression model selection process, we represented the summary SES measure as a continuous, piecewise linear function that has one join point at zero and is constrained to have zero slope when the PCA value exceeds zero.

Model selection by backwards elimination, starting with the 8 variables identified through our random forests analyses (see above), identified length-of-night, SES, and age as the only significant predictors of aMT6s (Table 3). Levels of aMT6s declined with older age and increased with longer nights and higher SES. Among the subset of women over the age of 55 years, SES was not a significant predictor of aMT6s while length-of-night and age became stronger and more significant predictors. Overall, these models explained very little of the variance in aMT6s (*R*^2^ for all women = 0.03; *R*^2^ for women > 55 years = 0.07).

**Table 3:** **Regression coefficients (β) for final model estimating log**_**2 **_**creatinine-adjusted aMT6s**

	**Variable**	**β, standard error**	**t statistic (2-sided p-value)**
All Subjects	Length-of-night	0.1081, 0.0612	1.76 (0.08)
	Age	−0.0113, 0.0066	−1.79 (0.09)
	SES	0.1375, 0.0637	2.16 (0.03)
Ages > 55 years	Length-of-night	0.2341, 0.0963	2.43 (0.02)
	Age	−0.0284, 0.0152	−1.87 (0.06)

Although the outdoor LAN variable was not selected through our stepwise model building approach, because it was of primary interest to us, we evaluated it separately in age-adjusted regression models and found it to have a very small inverse association with aMT6s that was not significant in either the full study population (*β* = −0.0028, se = 0.008, p = 0.73) or among those over age 55 (*β* = −0.0062, se = 0.012, p = 0.62).

## Discussion

Overall, results from our study failed to identify factors that substantially predict urinary measures of aMT6s. The only factors that were significantly related to aMT6s levels were age, length-of-night, and SES and together they explained very little of the variance in aMT6s levels. Nevertheless, our study does provide some important findings relevant to future investigations of health outcomes related to melatonin and/or LAN exposures.

There is increasing interest in the use of satellite imagery data to evaluate potential effects of outdoor environmental light pollution in wildlife and in human populations, including a number of studies that have investigated potential links to cancer incidence [20-22]. Our analyses demonstrated that the widely-available low-dynamic range annual satellite imagery data are insufficient for distinguishing areas with differing LAN values within suburban and urban areas. Since many health outcomes, including many cancers, are more common in urbanized areas [23-27], use of the low-dynamic range satellite data will likely be inadequate for investigating LAN exposures within such areas. While the high-dynamic range data are currently only available for 2006, efforts are underway to develop such high-dynamic range data for other years [28]. Researchers interested in this topic would be prudent to pursue such data.

The other novel finding generated by our analyses was the significance of neighborhood SES. Our analysis showed that aMT6s concentrations tended to be low among women living in the lowest SES areas and increased with greater neighborhood SES up to a point after which the concentration leveled off and possibly declined slightly at the very highest levels of SES. Furthermore, this relationship, while statistically significant in the full study population, appeared to be of lesser importance in the women over age 55. Overall, very little is known about how melatonin varies by SES. Similar to our findings, a study of approximately 200 Canadian women reported that urinary measures of aMT6s were lower among the lowest educated women (i.e. high school diploma or less), highest in the middle category of education, with a slight decrease in the highest educated group [29]. In contrast, Burgess and colleagues in their evaluation of predictors of salivary measures of melatonin found education was not a significant predictor [30].

Interpretation of our findings on SES can only be speculative. It is possible that the neighborhood measure of SES is serving as a proxy for a number of behavioral and/or anthropomorphic factors that influence melatonin levels. We did, however, incorporate variables in our model selection process for many of the most important of these factors including alcohol consumption, smoking, BMI, use of exogenous hormones and other medications, coffee consumption, and physical activity – none of which were independent and significant predictors of aMT6s. Another possibility is that low SES neighborhoods may have environmental stressors (e.g., noise, crime) other than light pollution that we did not measure in our study but could disrupt circadian rhythms and lead to lower levels of aMT6s. Given that a number of the health outcomes of high interest with respect to circadian disruption display strong SES-related risk gradients, research into other features of the built environment represents an important area for future inquiry.

In addition to SES, age and length-of-night were the only other significant predictors of aMT6s identified in our analysis. These findings are generally consistent with the limited body of literature on this topic. Two studies have reported seasonal differences in melatonin levels with higher levels associated with greater length-of-night [15,17] and lower levels in the summer months [17], although two other studies reported no relationship with month [18] or season [31]. Most studies have reported declines in melatonin levels with increasing age, albeit most have reported this relationship to be linear [15,17,18,31-33]. Consistent with our results, two studies noted more dramatic age-related declines among older individuals [34,35]; conversely, a few other studies suggested that the age-related declines in melatonin are greater, or are limited to, early adulthood and then level-off [18,36-38]. Measurements of melatonin in older adults living under controlled light–dark conditions in one study reported no age-related declines in melatonin levels, leading the authors to suggest that changes in sleep behaviors might be responsible for the age-related declines reported in other observational studies [39]. In our study, however, we observed no differences in sleep duration by age group (*data not show*). Our results, in the context of the somewhat conflicting literature on this, underscore the need to carefully consider the shape of the age-related risks when conducting health-related research on this topic. The fact that we observed a shift in the relationship between age and aMT6s around age 55 suggests this may be especially important when evaluating health risks in women, whose risks often change in their early- to mid-50s after menopause. This may be particularly relevant to breast cancer which exhibits a different constellation of risk relationships in pre- and post-menopausal women and for which LAN exposures have been postulated to be a potential risk factor.

Our evaluation of outdoor LAN, which showed a very modest inverse, but not statistically significant, relationship to aMT6s concentrations, was hindered by a number of obstacles. While we used the best satellite imagery data available to objectively estimate outdoor LAN values, because the high-dynamic range data was only available for 2006, the LAN estimates were not temporally congruent with the urine in which the aMT6s was measured (collected in 2000). An examination of the low-dynamic range data for all years spanning this time period (2000–2006), however, suggested relative stability in LAN values over this time for the study area. Furthermore, the Spearman rank correlation between the 2000 low-dynamic range data and the 2006 high-dynamic range data was 0.96.

Another reason for the lack of an association seen between outdoor LAN and urinary measures of aMT6s could be that while the satellite imagery data may be an adequate predictor of outdoor ambient light, it may not reflect light exposures experienced at night when participants are likely to spend the majority of their time indoors. Intervening factors, such as the use of blinds/curtains, time spent indoors, other sources of indoor light, etc. all are likely contributors to LAN exposures. The importance of this is underscored by findings from a recent study that compared estimates of LAN from satellite imagery data to calibrated photometric measurements obtained from personal monitors and reported that satellite imagery data did not correlate with personal photometric measurements [40]. Thus, it is important that studies aimed at evaluating outdoor LAN exposures do so with full consideration of these other factors.

Finally, there are a number of limitations in the estimates of our melatonin levels that are worth noting. The urine specimens were collected over a 24-hour time period. This precludes our ability to examine the timing of peak melatonin concentration which is likely an important factor in circadian disruption [13]. Furthermore, while there is a good deal of evidence that first morning urine captures peak night-time melatonin excretion, the degree to which 24-hour urine specimens reflect this is not well-documented [13,31]. The aMT6s assay, however, is a well-validated biomarker, demonstrating good correlation with serum melatonin levels [13], and sufficient stability over time [13,15,31,41,42] to serve as a reasonable estimate of chronic levels.

## Conclusions

There is growing evidence that circadian disruption, mediated by alterations in melatonin secretion driven by night-time light exposures may play an etiologic role in a large array of diseases. The use of satellite imagery data to estimate ambient measures of LAN offers an innovative and inexpensive source of data to test emerging hypotheses on this topic. The results from our analyses highlight some important cautionary limitations to the use of such data as well as point to some additional avenues of pursuit, including the elucidation of factors associated with neighborhood SES that may play a role in circadian disruption.

## Methods

### Study participants

Study participants consisted of 303 members of the California Teachers Study (CTS) residing in the San Francisco Bay Area, who as part of a special substudy conducted in 2000, completed a self-administered questionnaire, an in-person interview, and supplied a 24-hour urine sample. The CTS is a prospective cohort study initiated in 1995, consisting of 133,479 female professional California school employees for whom extensive information has been collected through a baseline questionnaire and three subsequent mailed questionnaires [43]. Members of the special substudy included a random sample of 528 CTS participants who were ≤ 85 years old when the cohort was established in 1995, and who resided in the substudy area (comprised of western Alameda, Santa Clara, San Mateo, Santa Cruz, Monterey, and northern San Benito counties). Forty-four (8%) of these women were not contacted because they had died, moved out of the substudy area, or could not be located. Of the 484 women invited to participate, 328 agreed, of whom 303 provided a 24-hour urine sample. Further details of the special substudy can be found elsewhere [44]. The use of human subjects was reviewed and approved by the California Health and Human Services Agency, committee for the Protection of Human Subjects and the Cancer Prevention Institute of California’s Institutional Review Board.

### Data and specimen collection

#### Specimen collection

Participants were given urine collection kits and instructed to collect all urine produced over the next 24-hour period. Participants were asked to use cold packs to keep the sample chilled and, if willing, to refrigerate it during the 24-hour collection period. After 24-hours, staff collected and stored the samples at -20° Celsius until they could be delivered to our laboratory (within 2 weeks) where it was thawed, aliquoted into 10 mL vials, and frozen at -70° Celsius. Prior to aliquoting, total urine volume was measured and recorded.

#### Laboratory methods

Urinary concentration of aMT6s was measured by Pacific Toxicology Laboratory (Chatsworth, CA, USA) using Bühlmann aMT6s enzyme-linked immunosorbent assay (ELISA) kits purchased through ALPCO (Salem, NH, USA). The aMT6s ELISA is a competitive immunoassay using a capture antibody technique that has a lower detection limit of 0.8 ng/mL and an analytic high sensitivity limit of 40 ng/mL. The functional sensitivity of the assay was 1.5 ng/mL, while the intra-and inter-assay precision were 7.1% and 11.9%, respectively. Urinary creatinine levels were obtained from laboratory assays using a modified-rate Jaffe method conducted as part of a prior analysis by the University of Alabama, Birmingham in 2000. aMT6s levels were creatinine-adjusted by dividing the aMT6s concentration by the creatinine concentration.

Four samples were below the detection limit and five samples exceeded the analytic high sensitivity limit (3% of samples total). Using PROC MI with a TRANSFORM LOG(var) statement in SAS 9.3 we imputed the values below and above the limit of detection, setting range parameters of 0–0.8 ng/mL and 40–60 ng/mL respectively. The upper range of 60 ng/mL for those samples above the analytic high sensitivity limit was based upon upper levels observed in 24-hour samples from women in a previous study [18]. We used the first imputation to assign values of aMT6s to these nine participants.

#### Outdoor environmental LAN data

Information on outdoor exposures to ambient LAN was derived from nighttime satellite imagery data obtained from the U.S. Defense Meteorological Satellite Program’s Operational Linescan System (DMSP-OLS) [45]. This database contains annual composites, made after excluding the outer quarters of the satellite swath, light from the sun and moon, glare, clouds, and atmospheric lightning. Ephemeral events, such as fires, are also discarded. While these images capture only a fraction of the light originating from the earth’s surface, they accurately represent the relative levels of nighttime illumination at ground level [45]. The imagery data is georectified to a 30 arc-second grid (equivalent to approximately one-square kilometer). Two kinds of radiance information were available: adaptive gain, low dynamic range data; and fixed-gain, high dynamic range data. The low dynamic range data are available for multiple years, but consist of unit-free (called DN, or “Digital Number” units) data with a dynamic range of six bits, i.e., integers ranging from 0 (background noise) to 63. The fixed gain, high dynamic range data, is available only for 2006 and is obtained by combining information from several fixed-gain sensors. It consists of floating point numbers (“scaled radiance” units) that, if needed, can be scaled by 1.51586 × 10^-10^ to obtain data that are watts/sr/cm^2^ (see the F16_2006_radiance_readme.txt file in reference [45] for caveats with respect to this scale factor). Such scaling was *not* performed for these analyses. Residences from study participants were assigned the average nighttime radiance value for the grid cell in which they were located using spatial analysis tools available in the raster, rgdal, and sp packages within R [46]. Initially we assigned to residences both the low-dynamic range data for 2000 and the high-dynamic range data for 2006.

As an additional measure of LAN we generated a “length-of-night” variable, created by linking the date of urine collection with daily sunrise and sunset data for San Francisco for the year 2000 obtained from the Naval Observatory [47]. From these data we calculated the hours of darkness on the day of urine collection.

#### Anthropomorphic and behavioral factors

Information on anthropomorphic and behavioral factors of interest was derived from the CTS surveys. Factors were considered as potential predictors for these analyses based on a review of the limited literature on this topic [15-18,31-35,37,38,42,48-51] and included: age, race/ethnicity, body mass index (BMI), height, parity, number of live births, age at first full-term pregnancy, pack-years of smoking, alcohol consumption, oral contraceptive use, hormone therapy use, menopausal status, strenuous physical activity, average hours of sleep per night, age at menarche, coffee consumption, and the use of aspirin, calcium blockers and antidepressants.

#### Sociodemographic data

Information on neighborhood socioeconomic characteristics and urbanization were derived from U.S. Census 2000 data. We used the ArcGIS, version 9.2 (ESRI, Redlands, CA, USA) to geocode the participants’ home addresses to both a latitude/longitude and to a Census 2000 block group. Data on socioeconomic status (SES) included: percentage of adults over age 25 years having completed a college degree or higher; percentage of adults without a high school degree; income (median family income), occupation (percentage of adults employed in managerial/professional occupations), and poverty (percentage of population below the poverty line). The degree of neighborhood urbanization was characterized as either urban/suburban or rural, based on a previously-developed algorithm using a combination of census block group characteristics, details of which can be found elsewhere [26].

#### Statistical methods

Given the extensive information collected on potential predictors of melatonin levels, our initial approach was to use Breiman’s random-forest-based (RF) variable importance measures, as described by Lunetta [52] to identify the most important predictors of log_2_ transformed, creatinine adjusted aMT6s from the large number of factors for which we had information. (The aMT6s levels were highly skewed to the right, necessitating the log_2_ transformation.) This approach allowed us to compose a list of candidate variables, ordered by their relative importance, taking into account both multiple testing and potential interactions. The top ranking variables were then considered in linear models to further quantify predictors of aMT6s concentrations. Final model selection via R’s stepAIC function was chosen in a backwards elimination process using the Akaike information criterion (AIC) to quantify the model fit.

We first focused on determining the most appropriate model specification for age. A plot, obtained by fitting a Generalized Additive Model (GAM) with an intercept and a spline term for age, demonstrated a decrease in aMT6s with increasing age, with a possible downward curvature at age 55. An analysis of variance to compare null, linear, and quadratic linear models for the regression of the creatinine adjusted aMT6s concentrations on age, however, indicated that the best fitting model was one with age modeled as a simple linear term. Because restricting our analyses to women over age 55 resulted in a much stronger and significant linear effect for age than observed in the full study population, we conducted our subsequent analyses on the full study sample, as well as among women over the age of 55. To address the high degree of collinearity between the SES variables, we conducted a principal components analysis (PCA) to create a composite measure of SES based on the five individual components as described above. The loadings of the first principal component were then used as a composite measure in the random forests and regression models.

## Abbreviations

aMT6s: 6-sulftoxymelatonin; LAN: Light at night; CTS: California Teachers Study; SES: Socioeconomic status; se: standard error.

## Competing interests

The authors declare that they have no competing interests.

## Authors’ contributions

SH participated in the design and coordination of the study and drafted the manuscript. DON obtained the satellite imagery data, assisted with the development of the GIS-based measures of SES, and oversaw the statistical analysis. EG conducted QA/QC on the laboratory data and assisted in the data analysis. RG assisted in the development of the study design and coordinated the laboratory assays. AH geocoded the residential addresses and assisted with assigning GIS-based measures to residences. PR participated in the design of the study and helped draft the manuscript. All authors read and approved the final version of the manuscript.
